# From System-Wide Differential Gene Expression to Perturbed Regulatory Factors: A Combinatorial Approach

**DOI:** 10.1371/journal.pone.0142147

**Published:** 2015-11-12

**Authors:** Gaurang Mahajan, Shekhar C. Mande

**Affiliations:** National Centre for Cell Science, Pune, India; Semmelweis University, HUNGARY

## Abstract

High-throughput experiments such as microarrays and deep sequencing provide large scale information on the pattern of gene expression, which undergoes extensive remodeling as the cell dynamically responds to varying environmental cues or has its function disrupted under pathological conditions. An important initial step in the systematic analysis and interpretation of genome-scale expression alteration involves identification of a set of perturbed transcriptional regulators whose differential activity can provide a proximate hypothesis to account for these transcriptomic changes. In the present work, we propose an unbiased and logically natural approach to transcription factor enrichment. It involves overlaying a list of experimentally determined differentially expressed genes on a background regulatory network coming from e.g. literature curation or computational motif scanning, and identifying that *subset* of regulators whose *aggregated target set* best discriminates between the altered and the unaffected genes. In other words, our methodology entails testing of all possible regulatory *subnetworks*, rather than just the target sets of *individual* regulators as is followed in most standard approaches. We have proposed an iterative search method to efficiently find such a combination, and benchmarked it on *E*. *coli* microarray and regulatory network data available in the public domain. Comparative analysis carried out on artificially generated differential expression profiles, as well as empirical factor overexpression data for *M*. *tuberculosis*, shows that our methodology provides marked improvement in accuracy of regulatory inference relative to the standard method that involves evaluating factor enrichment in an individual manner.

## Introduction

The availability of high-throughput technologies in recent years has made it possible to track the dynamics of a cell’s functional organization on the whole genome level [1,2]. The profile of genome-wide expression together with knowledge of the heterogeneous network of molecular interactions determines the cellular phenotype, and holds the potential for providing insights into how a cell adaptively responds to environmental cues [2–5]. Functional genomics experiments such as those based on microarrays or RNA deep sequencing are a rich source of information about the cellular milieu, and provide a starting point for generating causative hypotheses about biological mechanisms [6–9]. A question that routinely needs to be addressed in large scale expression studies is the identification of key regulatory pathways underpinning co-expressed, or differentially expressed genes. Transcription rates are controlled in part by a complex network of regulatory interactions involving DNA-binding transcription factors (TFs) and *cis/trans* acting regulatory sequences distributed throughout the genome [10–13]. Changes in the functional activity or expression of one or more of these proximally acting regulatory proteins–possibly representing consequences of signaling events initiated farther upstream–can directly reshape the transcriptome. This could describe a wide variety of situations, from the response of a cell to drug, to the comparison between two phenotypically distinct cell types, to even the difference between normal and diseased states. The inference of a set of ‘perturbed’ regulators is an initial and important step towards arriving at a broader mechanistic interpretation of any genome-scale profile of altered expression.

*De novo* approaches seeking to discover shared factor-binding DNA sequence motifs within the promoter regions of altered genes provide a rational starting point [14,15]. Such regulatory information for the genomes of many species continues to accumulate at a rapid rate from ChIP-seq experiments as well as low-throughput studies [16–23]. Databases providing experimentally determined/predicted transcription factor binding sites, TF motif profiles, and even meta-network information curated from literature evidence [24–31] are routinely available now, and could be usefully exploited by experimentalists interested in understanding differential TF activation in specific contexts. Towards this end, many bioinformatics tools have come up in recent times that facilitate such regulatory analysis. These methods [32–43] share the common denominator that an input list of genes specified by the user, e.g. coming from a microarray study, is overlaid on a pre-specified background regulatory map connecting transcription factors to their target genes. This input list might represent the genes detected to be significantly differentially transcribed in a case vs control comparison of genome-scale expression. In order to deal with the noisy nature of the data, some appropriate statistical test is applied to each TF in the back-end network to determine a statistically significant association, or over-abundance, between the targets of the TF and the input gene list, relative to the overall genomic background. (In the rest of the paper, our use of the terms ‘enrichment’ or ‘association’ in the context of TFs will be intended to mean that the target set of that TF is enriched for significantly differentially transcribed genes.) Depending on the over-representation p-values computed, a prioritized list of candidate regulatory factors likely to be most relevant for interpretation of the user’s data is thereby generated.

A few examples of such applications are noted here. ChIP Enrichment Analysis (ChEA-X) is one such popular tool that leverages a curated database of ChIP-seq profiles from mouse and human experiments to compute over-represented target sets using Fisher’s exact test of significance [32,33]. Two related applications, Kinase Enrichment Analysis (KEA) and Expression2Kinases (X2K), are methodologically similar but go a step further and, by additionally exploiting curated data on kinase-substrate relationships, suggest signaling pathways highlighted by input lists of altered genes [34,35]. ENCODE ChIP-Seq Significance Tool is a web-based interface which allows users to mine a back-end comprised of mouse and human TF binding site data generated as part of the ENCODE series of experiments [36]. Hypergeometric test is applied to score individual transcriptional regulators for significant association with the input list of genes. This test is similarly the basis for TF enrichment analysis implemented within the RENATO [37] and WebGestalt [38] tools. Other utilities such as Whole-Genome rVISTA [39,40], Promoter Integration in Microarray Analysis (PRIMA) [41], Cis-eLement OVERrepresentation (Clover) [42] and Relative OVER-abundance of cis-elements (ROVER) [43] work instead with the binding site motifs of known TFs, represented as position weight matrices (PWMs), information about which can be found compiled in resources such as TRANSFAC, JASPAR, HOCOMOCO, UniPROBE etc. [27–31]. Despite differing in the actual criterion applied for assigning target genes to every regulator, which is based on scanning of promoter sequences for high-scoring motif matches, they all nonetheless follow the common theme that over-abundance scores relative to the genomic background (i.e. p-values) are calculated for each regulatory motif *separately* against the *entire* list of input genes. Moreover, the null background implicitly assumed in all the above approaches is essentially one of no association, corresponding to a random distribution of significantly altered genes over the genome.

**Fig 1:**
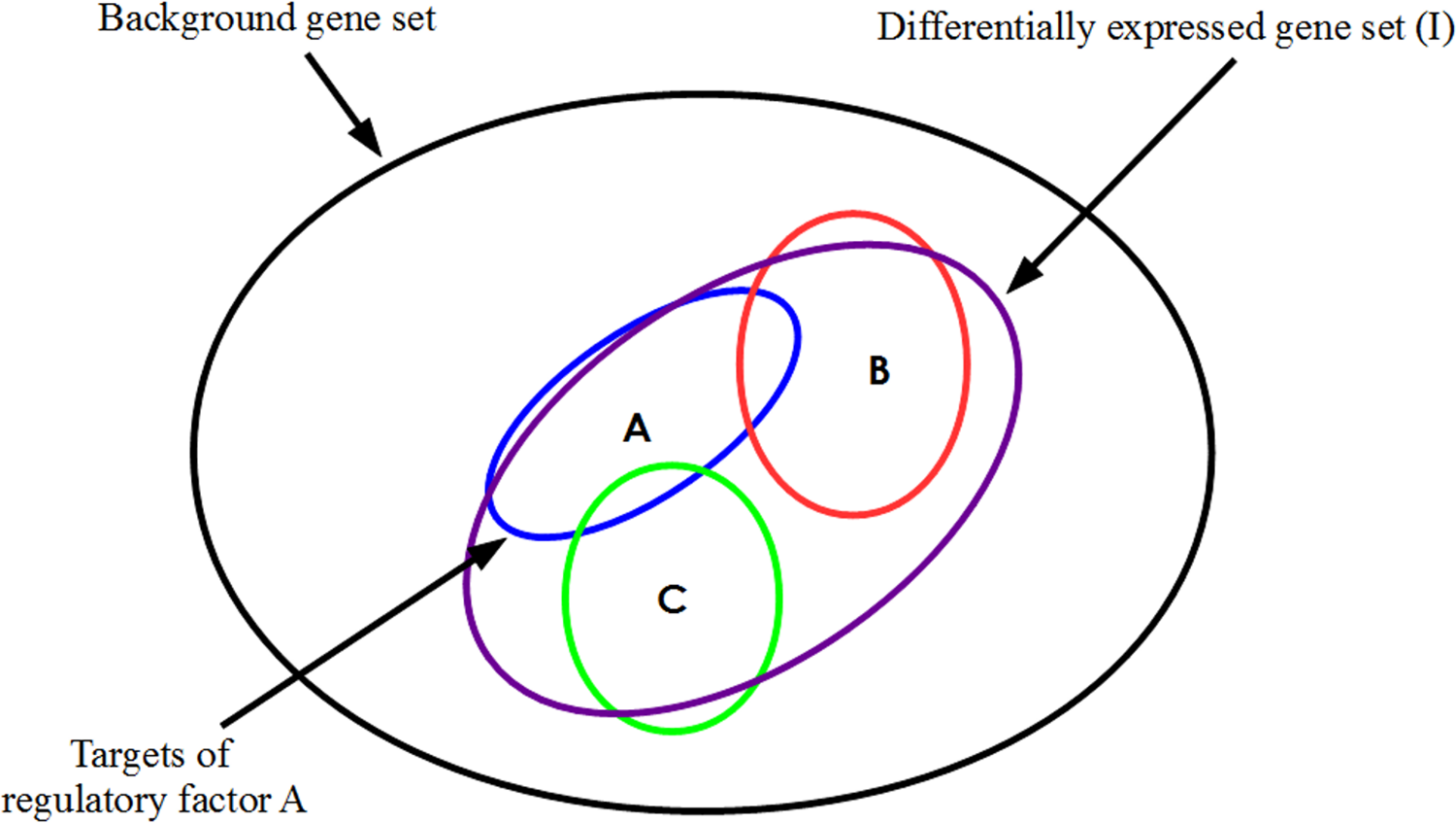
Differential expression profile overlaid on a transcriptional regulatory network. Venn diagram illustrating an example situation involving three transcription factors A, B and C each of which targets a distinct group of genes. Each target set also has an overlap with the input set I, which represents the genes detected as significantly differentially expressed in a case v/s control comparison of transcriptome data.

When evaluating individual TFs for association with a large significantly differentially expressed gene set detected in a transcriptomic experiment [44–46], it is worth noting that the above methods are likely to work well when one or only a very small number of TFs have been differentially activated. On the other hand, if a gene list represents the collective consequence of perturbing multiple regulators, then it is conceivable that individual TFs may well fail to show up as statistically significant upon application of one of the tests previously mentioned. The hypothetical situation in Fig 1 serves to illustrate this point. It is not hard to imagine a case where the target set of factor A, or B or C by itself may not show statistical association with the full input gene set I. The effect of other regulators acting concomitantly raises the possibility that any one TF may fall short of achieving separation between the altered and unaltered genes, when assessed in terms of the corresponding p-value of enrichment. Thus, the deductions made about relevance of individual transcription factors for alterations in gene expression might be inaccurate.

From Fig 1 it also follows that aggregating the targets of A, B and C together would yield a significant association of this *union* with the differentially expressed gene set. This suggests an alternative approach to delineating a set of immediately upstream transcriptional regulators causally underlying an altered expression profile. In the present work we propose a perspective which entails the testing of all possible *subsets* of TFs, i.e. the unions of their target genes, instead of just the target sets of individual TFs. In our opinion, this reformulation would appear to be a more natural and powerful approach to ascribing differential gene regulation to a set of perturbed transcription factors. A hypothesis for differential expression is therefore proposed by identifying the TF *combination* that best separates the differentially regulated genes from the unaltered genes derived from any high-throughput expression profiling experiment.

## Results

### The default approach to TF enrichment can lead to inaccurate inferences in an idealized setting

A microarray expression profile can be regarded as representing a combination of signal (*S*), the 'true' pattern of differential transcription resulting from altered TF activity, and noise (*η*) introduced by various sources which result in the occurrence of false positives/negatives. We begin our analysis by considering TF enrichment in the *η* → 0 deterministic limit, where all the gene targets of a randomly selected group of TFs are labeled as differentially expressed. This noiseless limit can be expected to provide an upper bound on the efficacy of any statistical enrichment test. We first assess the *default*, or standard, method of testing, which involves estimating over-abundance p-values for the target sets of *individual* TFs. As mentioned in the introduction, this is identical to the approach followed by several published tools currently in use for regulatory analysis [32–43]. What needs to be evaluated is how the p-value for every TF varies with the number of perturbed regulators, and the possible effects of target set size and the co-targeting of common genes on the ascribing of statistical significance.

**Fig 2:**
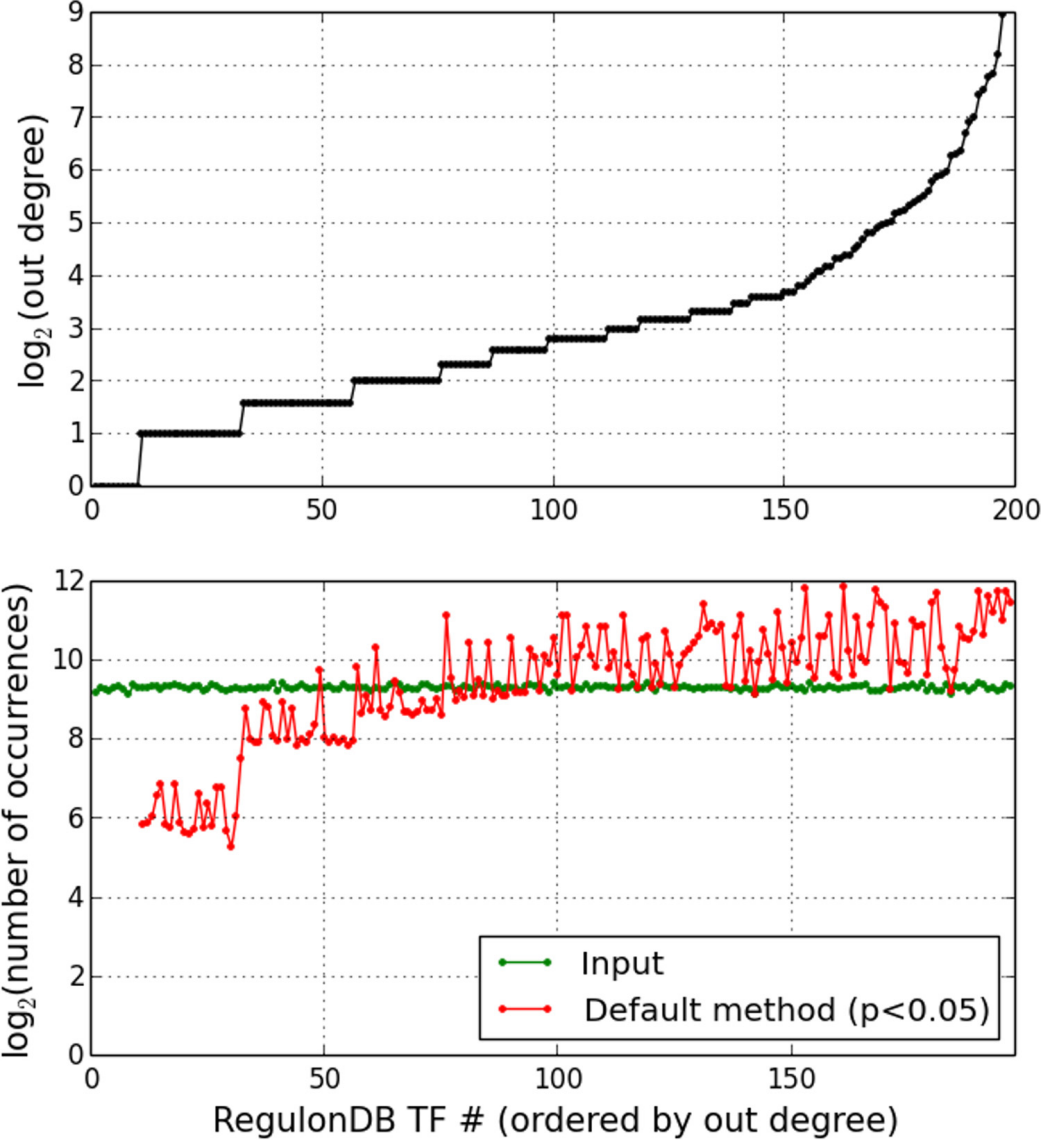
Transcription factor enrichment from simulated trials. Upper panel: The *E*. *coli* regulatory meta-network used contains 197 TFs, exhibiting a broad out-degree distribution. Lower panel: Distribution of transcription factor occurrences in the input and statistically enriched sets, obtained by aggregating 104 randomly generated binarized profiles in the noiseless limit.

**Fig 3:**
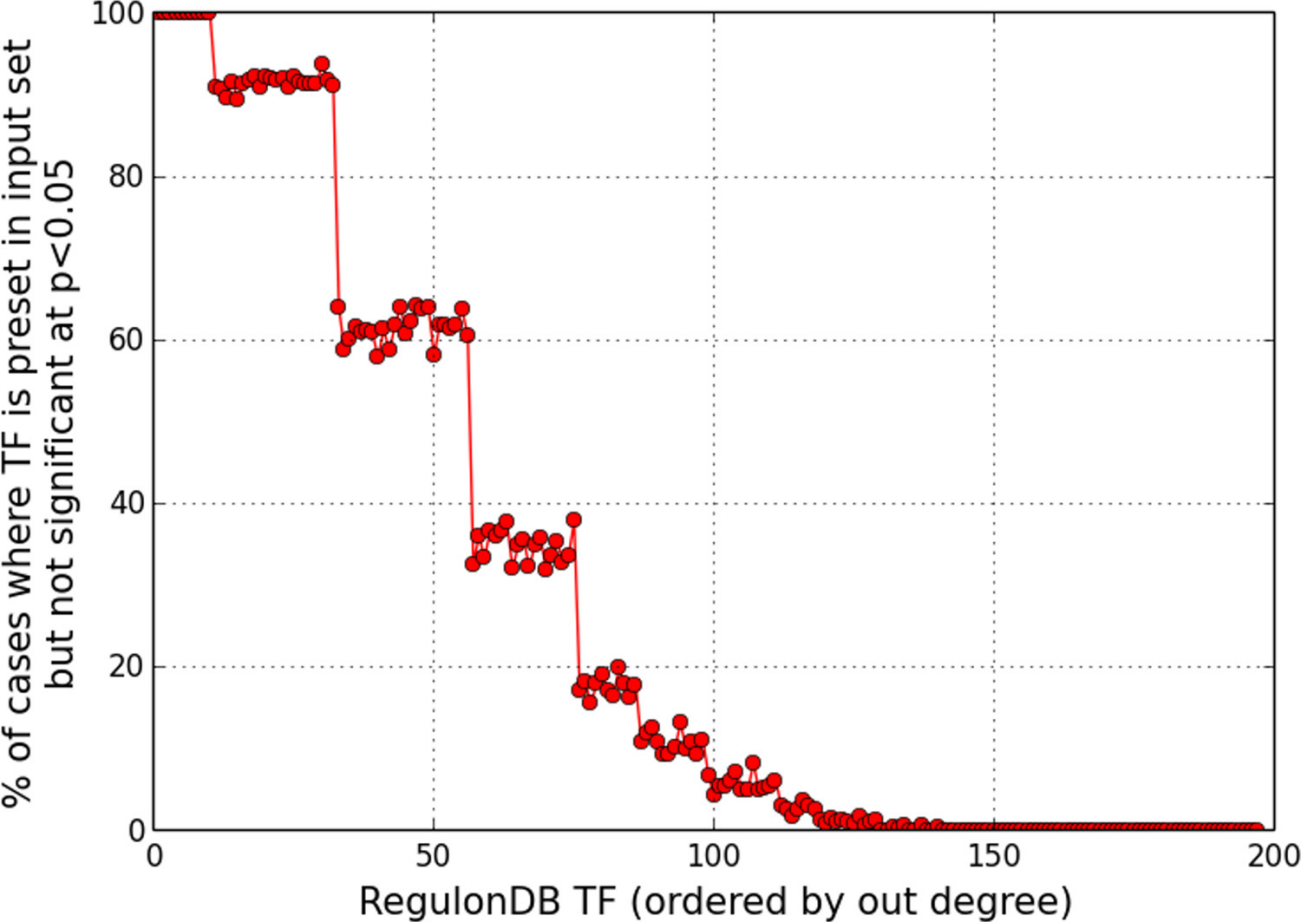
Under-representation of TFs in enriched set. TFs with smaller target set sizes may not always be statistically enriched, if multiple TFs are simultaneously ‘perturbed’ producing an extensively altered expression profile.

Figs 2 and 3 summarize the results for 10^4^ runs with simulated expression data on the *E*. *coli* RegulonDB transcriptional regulatory network [26]. The efficacy of the default method has been gauged in terms of the frequency distribution of TF occurrences in the enriched set. This is compared with the underlying 'input' TF distribution, which to the first approximation is uniform. Fig 2 displays the comparison for a p-value significance threshold of 0.05, with TFs being ordered according to their out-degrees along the horizontal axis. Two features that Fig 2 (lower panel) illustrates are the over-representation of the higher degree regulators in the output set on the one hand, and a dip in the frequencies of occurrence of the TFs with small target sets on the other. This can be seen by a direct comparison of the input (green) and enriched (red) distributions. Similar trend is also seen for other choices of the p-value threshold, in the range 0.01-1e-6. These features suggest that when multiple TFs are simultaneously perturbed, regulators with smaller target set sizes might fail to show statistically significant enrichment for the combined differentially transcribed gene set. Further, TFs which are not differentially activated, but co-target genes with other regulators, can also show a spurious association when evaluated by the hypergeometric test.

Fig 3 presents the above trend from a slightly different angle. The percentage of runs in which any given regulator is included in the input set, but whose target set does *not* show a significant over-abundance, is plotted against the ordered sequence of TFs ranked by increasing out-degree. This representation is consistent with the dip seen at the lower end in Fig 2 (lower panel), and brings out the possibility that, when evaluated one at a time against the combined set of *all* differentially expressed genes, TFs with smaller target sets might be missed out by the test for significance when regulatory activity of multiple factors is altered in concert. Thus, an artefactual under-representation of TFs may occur.

### Benchmarking reveals the best among three iterative search methods

We now explore testing combinations of TFs for association against the differentially expressed genes, instead of treating each TF separately, such that the combination which is found most consistent (in terms of minimum p-value for collective association) provides an alternative hypothesis for the altered regulatory activity. However, the infeasibility of exhaustively searching the space of possible combinations presents a practical stumbling block. For example, in typical bacterial networks, there are O(100) transcription factors [26,47,48]. The number of TFs targeting differentially expressed genes in typical genome-scale datasets would be of the same order of magnitude, requiring the computation of O(2^100^) or ~ O(10^30^) enrichment p-values. Restricting the biologically sensible combinations to not more than 20 TFs in size, still leaves about 100!/(20!*70!) possibilities to be tested, and even this number would be beyond the limitations of computational tractability. With eukaryotic gene regulatory networks and especially those for mouse or human, a factor of 10 increase over bacteria in the number of regulators to O(500–1000) is expected, based on standard regulatory meta-network datasets used in the literature [19–25]. This would translate into an even larger number of candidate combinations that would need to be tested to identify the best-fit hypothesis. We therefore seek a solution that is sufficiently close to the global minimum, through a computationally efficient heuristic approach.

**Fig 4:**
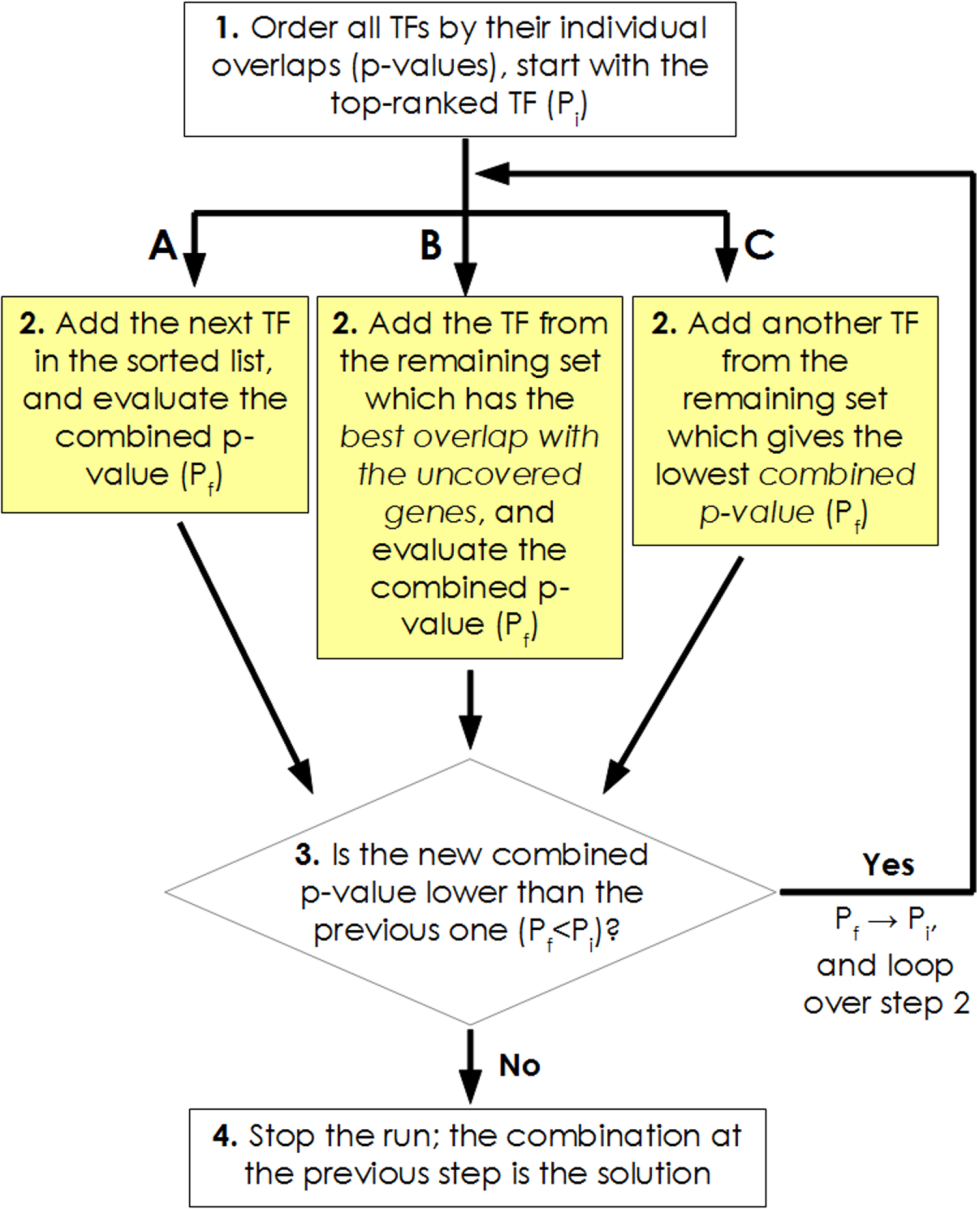
Schematic representation of the iterative search methods A, B and C for obtaining an approximation for the most predictive regulatory subnetwork. A, B and C differ only at step 2, indicated here in separate yellow boxes.

As the nonlinearity of the objective function sought to be minimized precludes the adoption of exact linear programming methods, we have tested three iterative search methods to efficiently arrive at such an approximation. These are described in the *Materials and Methods* section, and represented together as a flowchart in Fig 4. Method A is linear in the dimensionality of the search space, i.e. in the number of TFs tested, and the solution is built up by sequentially adding TFs in increasing order of their individual p-values. In the case of the Method B, it is easy to see that O(N) p-values have to be estimated at every iteration, because the differentially expressed gene set shrinks at every step as the genes already covered by the TFs selected up to that point are systematically eliminated from consideration. The origin of a quadratic runtime follows from this. The O(N^2^) scaling of Method C also arises from the fact that the p-values corresponding to the TFs not already included in the growing solution have to be recomputed at every update.

**Fig 5:**
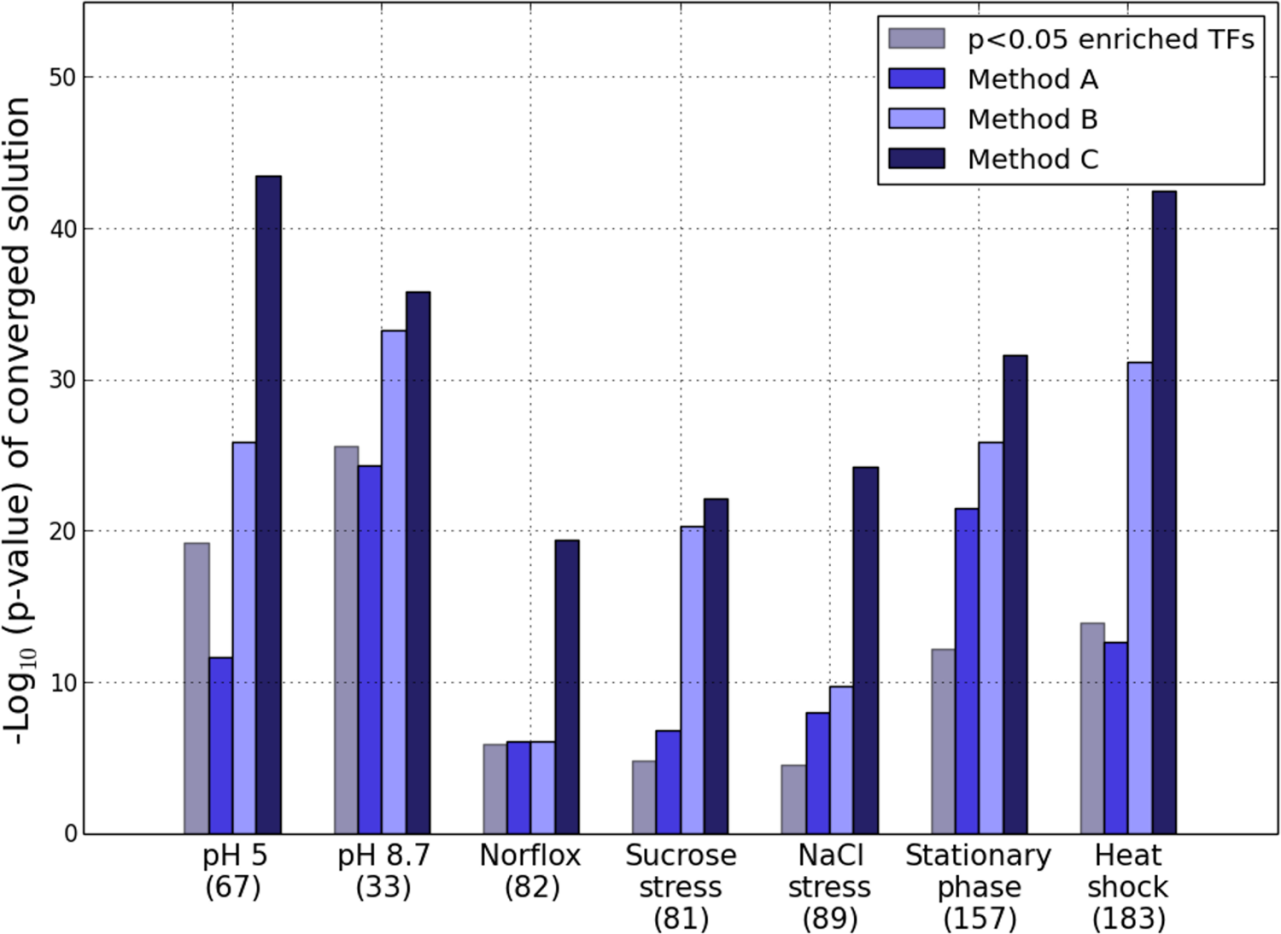
Benchmarking the iterative methods on GEO microarray datasets. Comparison of the iterative methods A-C to identify the maximally discriminative group of TFs, applied to 7 *E*. *coli* expression datasets retrieved from GEO. The number in brackets under each label corresponds to the number of TFs in the background network targeting at least one significantly differentially expressed gene each. For sake of comparison we also include the result for the default approach, which has been obtained by merging the target sets for all the TFs showing individual overrepresentation (at p<0.05 level), and estimating the collective p-value for this union.

Fig 5 displays the results for the combinations obtained by running each of the three heuristics, Methods A-C, on 7 GEO datasets [49–52]. This selection is not intended to be exhaustive in any way, but is representative of the microarray data available in the public domain, and serves to illustrate the salient features of our approach as well as some statistical properties of the search space. It is seen clearly that across all the 7 conditions analyzed, Method C outperforms the other two heuristics, yielding solutions with lower enrichment p-values. For reference, the combined overlap p-value obtained by merging the target sets of all individually enriched TFs (at p<0.05 level) is also shown, from which the improvement given by Method C is readily apparent.

In addition to identifying TFs that are not individually associated with the differentially expressed genes at p<0.05 significance level (S1 Table, column 2), Method C also increases the coverage of the differentially expressed genes (S1 Table, columns 3/4), even though both the approaches only assess the statistical association in terms of p-values.

A three-way comparison was also carried out on the larger M3D expression dataset [53]. Based on the set of perturbed genes identified in each condition, the associated upstream transcription factors were then inferred by applying the three approximations in turn. The results of this analysis are summarized in Table 1. We assessed the performance of Method C separately against Methods A and B. In line with the outcomes in Fig 5, we find that Method C displays improved performance overall. For instance, in over 98% of the experiments, Method C yields a significance p-value that is at least as small as the outcome of Method B. Similarly, in no experiment does Method A improve on the p-value yielded by the application of Method C. It may also be noted that these results are reproduced across three different choices for the expression z-score threshold (2, 2.5 and 3). Further, like before, an overall increase in the coverage of the differentially expressed genes by the identified TFs is observed (S1 Fig).

**Table 1:** Benchmarking iterative search methods on M3D expression profiles.

Pairwisecomparison (in terms ofp-values)	|z-score| > 2(# expts. = 466)	|z-score| > 2.5(# expts. = 438)	|z-score| > 3(# expts. = 396)
Method C > Method B	67.19	46.34	27.27
Method C > = Method B	98.92	99.32	98.73
Method C > Method A	92.49	77.63	68.18
Method C > = Method A	100	100	100
Method B > Method A	71.88	61.41	58.33
Method B > = Method A	96.13	97.26	98.23

Three-way comparison among the heuristics proposed, based on 466 experiments with *E*. *coli* compiled under M3D. Each column displays the results for a particular choice of the z-score cutoff applied for identifying genes with altered expression in every experiment. Numbers in brackets in the column headers are counts of those experiments in which at least one altered gene is targeted by a TF from the underlying network. All comparisons are percentages relative to the corresponding total number of experiments being considered. Performance of each method on every expression profile is quantified in terms of the collective over-representation p-value for the TF subset it converges to.

**Fig 6:**
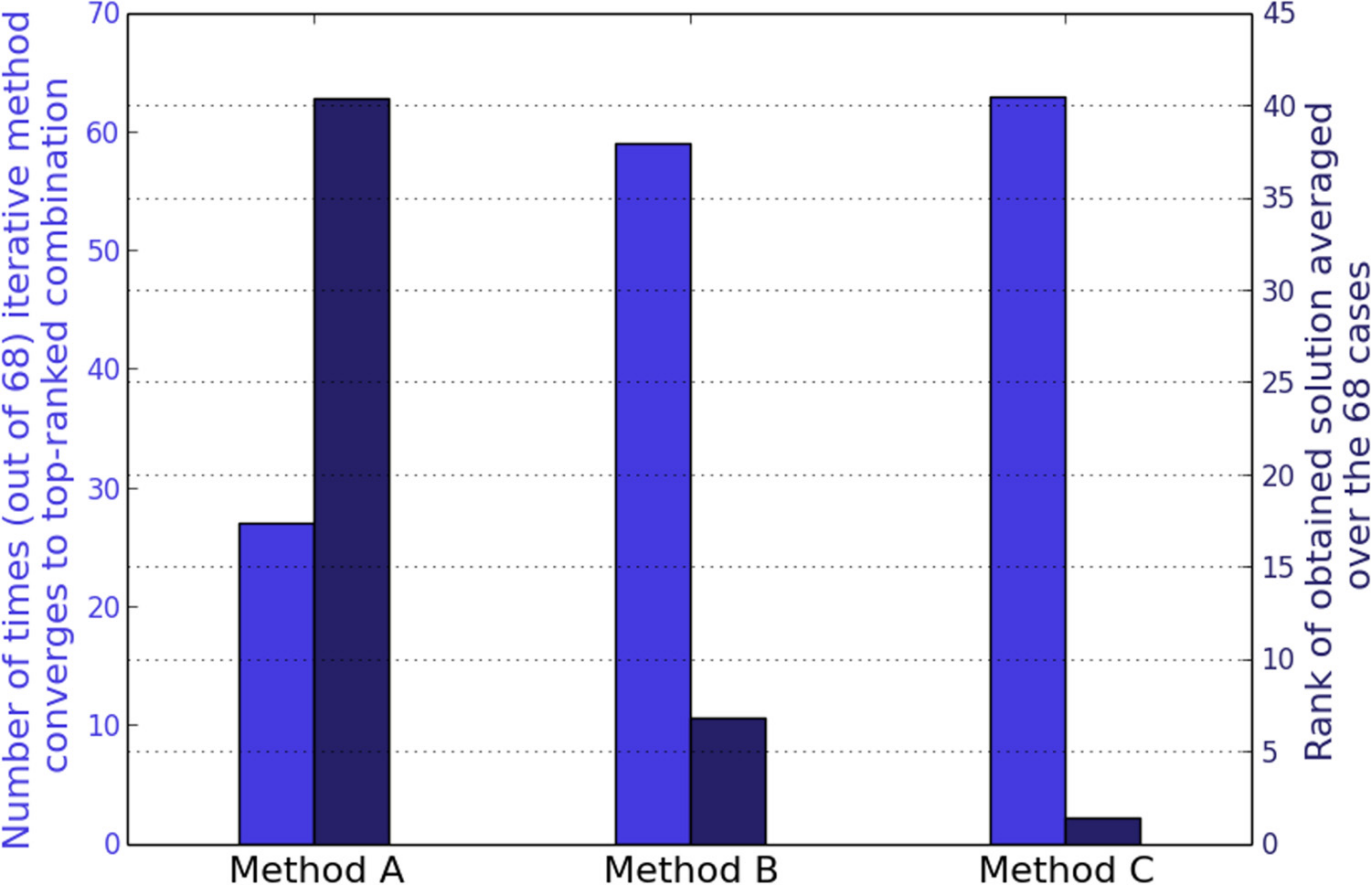
Benchmarking the iterative methods on M3D data for *E*. *coli*. Comparison of the three heuristics applied to a subset of M3D expression profiles where the manageable numbers of TFs make exhaustive enumeration possible (z-score threshold for significance of altered expression = ± 2.0). The light blue bars represent the number of times that each of the methods identifies the global minimum (left y-axis). The dark blue bars display the mean rank of the estimated solution in a list of all possible TF combinations sorted according to their combined p-values (right y-axis).

Out of the 466 experiments in total, with a z-score cutoff of 2.0, we found 68 conditions in which the differentially transcribed gene set sizes were small enough such that only ≤ 14 TFs overlapped with these genes. In these instances, it was possible to identify the exact solution by enumeration over all possible combinations, allowing for a direct comparison with the results of the greedy methods. Across these 68 cases, we have estimated the number of conditions in which each of the three methods yields a p-value equal to that of the globally top-scoring combination. In addition, the quality of the obtained approximate solution has been assessed by its rank in an ordered list of TF subsets representing the full search space. Fig 6 summarizes the results of this exercise, supporting our earlier conclusion about the better performance of the O(N^2^) Method C, which not only yields the true best p-value more often but is also able to get closer to the global minimum, as assessed by the average rank of the obtained solutions. Similar results were obtained with the more stringent expression z-score threshold of 3.0 as well (S2 Fig).

### Search space can be rugged with multiple local solutions

The heuristics considered above represent incremental approaches which only sample the local neighbourhood–comprising the current solution and adjacent combinations each differing by a single TF–at every update. Such local search might only yield a sub-optimal solution, rather than the global minimum, when multiple local minima coexist [54,55]. It is therefore of interest to ascertain the extent to which the exponential search space composed of all possible combinations of N transcription factors is rugged [55–57]. This has been done by estimating two measures: the autocorrelation of random walks and the number of local minima in the p-value landscape.

**Fig 7:**
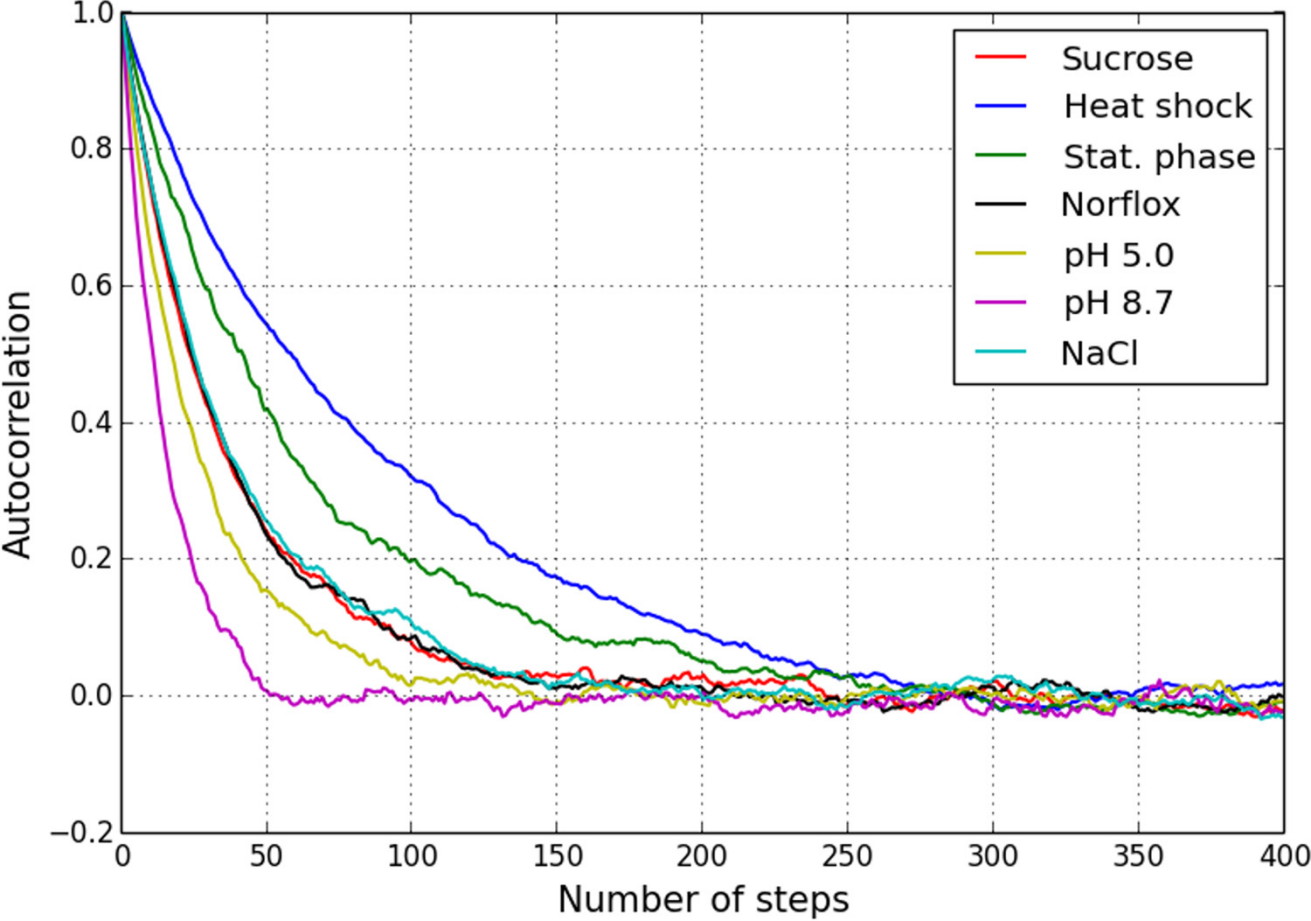
Autocorrelation along random trajectories in the search space for the seven *E*. *coli* microarray profiles. Each curve has been obtained by averaging over 5000 random walks starting from different initial combinations. All the cases show decay over O(N) steps, N being the number of TFs targeting at least one significantly differentially transcribed gene each (dimensionality of the search space).

Fig 7 shows the autocorrelation (Pearson correlation coefficient) of the combinatorial log p-value plotted as a function of the number of steps, for all 7 GEO datasets we ran the iterative methods on earlier. The autocorrelation has been estimated by averaging over 5000 random walks starting from different (arbitrary) initial configurations [54]. The decay over O(N) steps seen here is suggestive of a moderately rugged search space.

**Fig 8:**
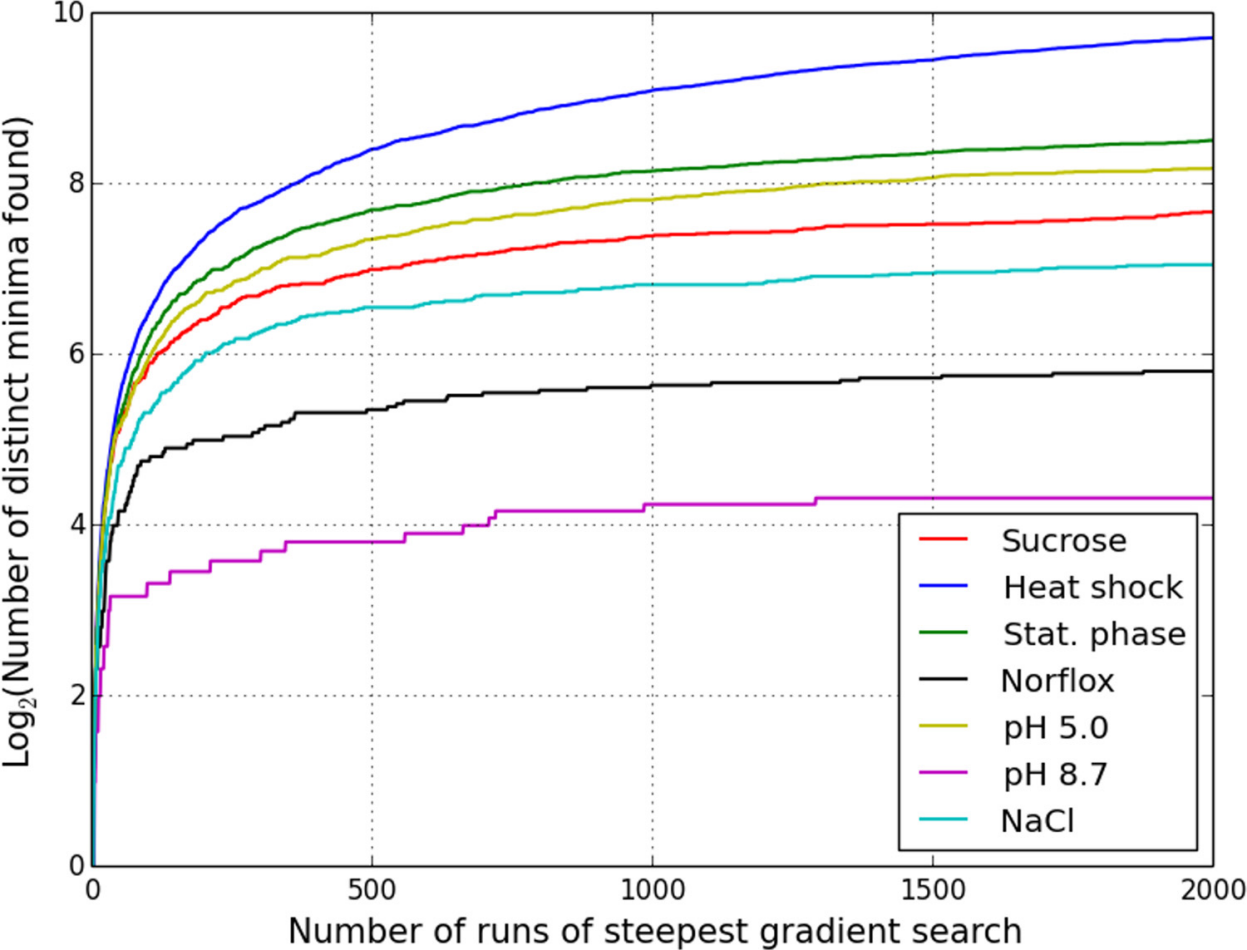
Ruggedness of problem search space for the seven *E*. *coli* microarray profiles. The number of local minima reached by steepest descent search as a function of the number of initial conditions sampled provides a statistical summary of the combinatorial search space.

The ruggedness has been further quantified by implementing steepest gradient search, and tracking the number of distinct local minima of the p-value attained as a function of the number of runs, starting from random initial configurations [54–57]. Every run converges to the minimum which lies closest to the starting configuration. The results of this exercise for all 7 conditions are shown in Fig 8. The number of minima sampled by steepest descent search initially grows with the number of runs, before beginning to flatten. The existence of multiple minima is indicative of a complex search space, requiring simple local search to be repeated a number of times with random restarts for adequate coverage. However, this is clearly an inefficient approach, because even a large number of runs does not ensure that *all* local minima would get sampled and the global minimum of the combined p-value identified with certainty. This is apparent from the monotonic but irregular curves in Fig 8, which illustrate the sporadic nature of the sampling of local minima by repeated steepest descent search.

We further note that the iterative Method C, while being a local search procedure, is initialized in a non-random manner, and only allows addition, but not deletion, of TFs at every update. We have compared the estimate yielded by Method C with the sorted list of p-values sampled by 2000 runs of steepest descent search. The result of this exercise, tabulated in columns 3 and 4 in Table 2, shows that Method C, at least for the examples considered here, provides a more efficient alternative to the more conventional local descent search with random initializations. Method C converges to the top-ranked p-value identified by gradient search method in 4 of the 7 conditions. Even in the case of the heat shock dataset where the rank is somewhat lower (84/840), the solution estimated by Method C still falls in the top 10% of the ordered list. The low probabilities in column 3, with the possible exception of the ionic osmotic stress (NaCl) example, additionally show that the minima Method C converges to are atypical, and the corresponding basins of attraction are restricted, both of which make it improbable that one run of steepest descent search with random initialization improves on the result of Method C.

**Table 2:** Summary of results for seven *E*. *coli* microarray datasets.

Experiment	Number (resp. %) of genes found differentially expressed (p<0.001, log fold change>1)	Fraction of runs of steepest descent search yielding a local solution at least as good as Method C	Rank of solution found by Method C in a sorted list of local minima sampled by steepest descent search	Log p-value from Method C (sig. z-score)	Minimum log p-value from steepest descent search	Minimum log p-value from SA
pH 5.0	176/4345 (4.1%)	0.06	1/291	-43.44(-12.9)	-43.44	-43.44
pH 8.7	92/4345 (2.1%)	0	1/20	-35.79 (-11.9)	-8.82	-35.79
Norfloxacin	312/4345 (7.2%)	0.027	1/56	-19.37 (-2.8)	-19.37	-19.37
Sucrose stress	277/4070 (6.8%)	0	1/205	-22.17 (-3.8)	-11.87	-22.17
NaCl stress	194/4070 (4.8%)	0.26	2/133	-24.24 (-5.1)	-24.32	-24.32
Stationary phase	2314/4453 (51.9%)	0.003	3/365	-31.65 (-6.4)	-32.16	-32.16
Heat shock	3139/4453 (70.5%)	0.1	84/840	-42.44 (-7.9)	-46.16	-46.16

Benchmarking of Method C v/s steepest descent search based on 7 microarray expression datasets describing *E*. *coli* stress adaptation. The latter has been run 2000 times with random restarts on each dataset. Also tabulated are the p-values obtained from the simulated annealing procedure, which provides an independent estimate of the global minimum.

**Fig 9:**
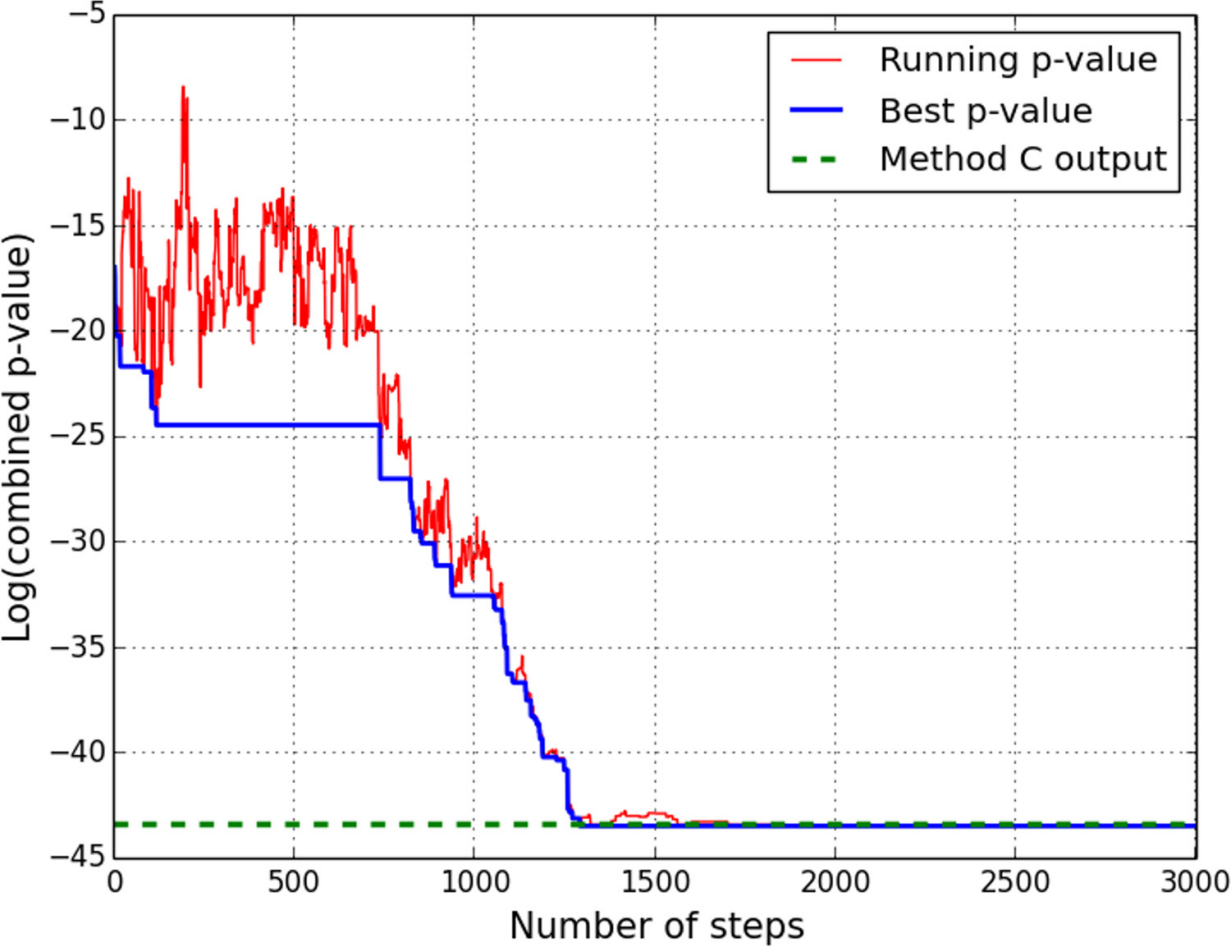
Example of simulated annealing search. A single run of SA stochastic search for the global minimum p-value, applied to the pH = 5 vs pH = 7 comparison microarray dataset. Sharp fluctuations in the red curve arise from the possibility of non-optimal updates, the propensity for which depends on the (gradually decreasing) annealing temperature.

In view of the considerable ruggedness of the search space besides the broad range of objective function values spanned by the local minima, we also implemented stochastic search via a simple version of simulated annealing [58]. This involved carrying out multiple independent runs of an exponential annealing schedule with different settings for the rate parameter *r*. Fig 9 illustrates the evolution of the p-value over one run of SA applied to the acid stress (pH = 5.0) dataset; the solution obtained by Method C is additionally shown for comparison. SA provides an independent objective benchmark against which to assess the results from the iterative methods. As summarized by columns 5–7 in Table 2, Method C converges to the presumably true global minimum estimated by SA in 4 of the 7 examples. A similar comparison was also made on the M3D dataset [53]. The expression z-score profiles were sorted according to the number of TFs targeting the significantly altered gene set, and the two methods were then applied to the subset of top 20 profiles, for which the search space dimensionality (overall number of TFs targeting the significantly altered genes) ranged between 110 and 140. In 11 (55%) cases, Method C yields p-values which match those obtained by the SA search.

A stochastic method like SA is generally more preferable in a rugged search space [58–61], but requires either very slow rate or multiple runs covering a broad range of parameter settings (as implemented here) to reliably converge to the global minimum, and either of these increases the running time. This may be a practical drawback when elaborate expression studies involving large number of conditions/time points need to be analyzed. The preceding comparison with stochastic and local searches taken together thus suggests that our iterative method C potentially provides a good balance between speed and quality of estimated solution. Its efficacy as evidenced by the examples considered here makes a case for its utility for estimating the maximally discriminative group of TFs from a binarized profile of gene expression changes.

### A randomization procedure provides a test for consistency between global expression changes and the underlying TF regulatory network

For all seven *E*. *coli* datasets considered, the correlation of the binarized differential expression profile with the underlying transcriptional regulatory network has been quantified in terms of a z-score for the collective log p-value for the TF combination yielded by Method C. As described in *Materials & Methods*, this z-score calculation is based on a negative control distribution of p-values, which is obtained by a randomization procedure that redistributes the differentially expressed genes over the network. The combinatorial search (iterative Method C) is run on each such randomized profile, and the set of log p-values obtained in this way define a baseline distribution. K-S test for normality [62] has been run on this sample of log p-values, and across all 7 examples it is found to be consistent with a normal distribution (p-value for null hypothesis > 0.2 in all the 7 cases). The estimated z-scores are listed in Table 2 (column 5), and range from z = -2.8 (norfloxacin) to z = -12.9 (acid stress). Large and negative z-scores are indicative of consistency between the empirical differential expression profiles and the background TF network adopted for drawing regulatory inferences, and hence provide support for the relevance of the identified TF subsets as a basis for the global transcriptional changes.

### The proposed approach leads to less biased, more accurate inferences of differentially acting TFs

**Fig 10:**
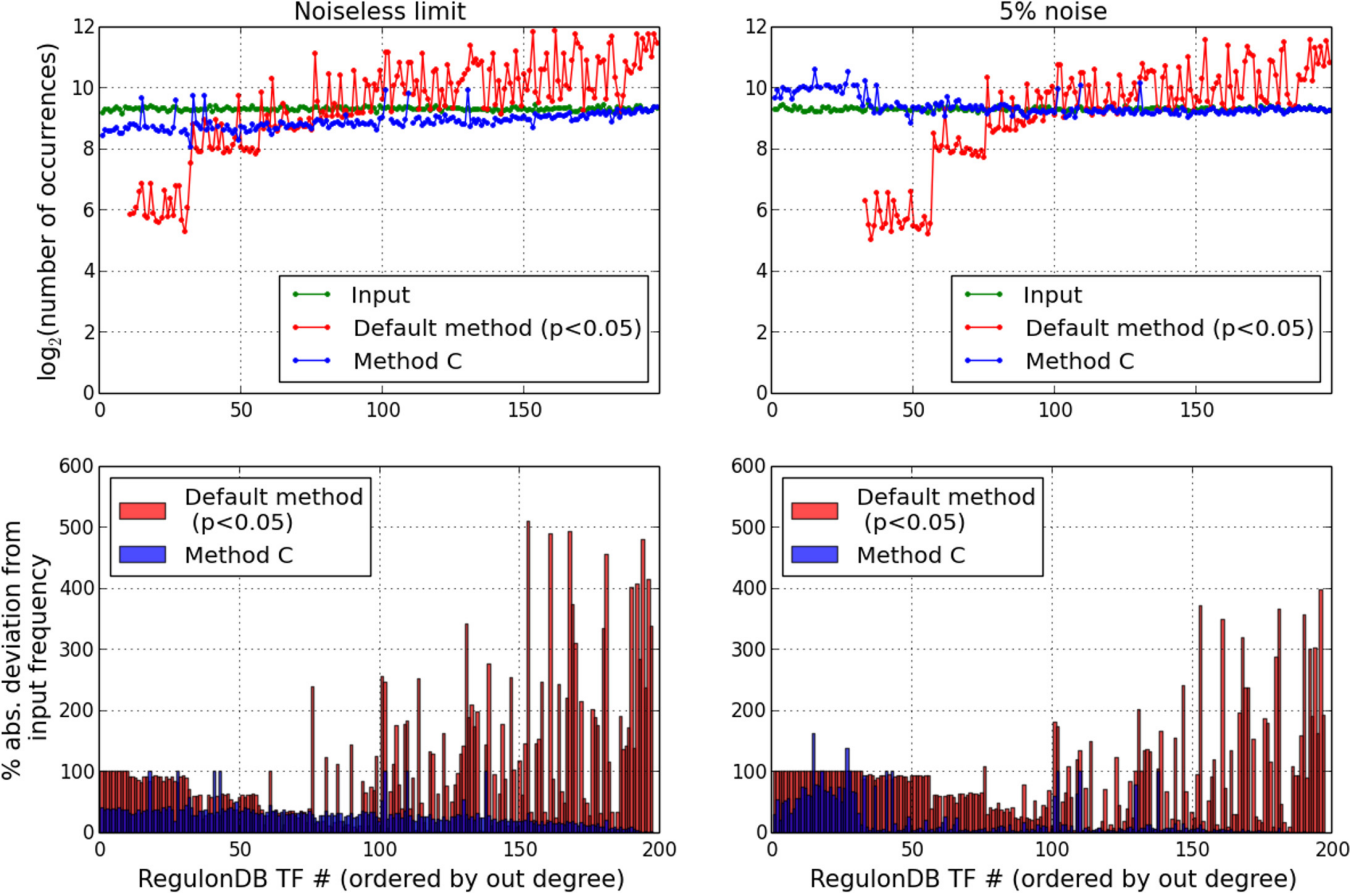
Comparison of the combinatorial approach (iterative Method C) with the default method on simulated trials. Distribution of TF occurrences (over 104 trials) in the noiseless limit which was introduced in Fig 2 (left panels), and with the addition of 5% misclassification rate (right panels). Deviations of the red and blue curves from the green profile in the top panels have been represented in terms of profiles of percentage differences in the bottom panels.

We return to the noiseless limit delineated earlier in *Results*, with random combinations of TFs being selected for perturbation and used as a basis for assigning genes to the altered/unaffected sets in a deterministic manner. We found that when statistical enrichment is assessed for every TF separately against the full set of differentially expressed genes, the sub-dominant TFs with smaller target sets get under-represented in the recovered set (based on adjusted p-values ≤ some threshold), while the global regulators tend to over-occur (Figs 2 and 3). We would like to compare this outcome with the results of the Method C which provides an approximation for the TF combination with minimum collective p-value. Fig 10 (upper left panel) displays the result for the distribution of TF occurrence frequencies across 10^4^ random trials. This plot is the same as in Fig 2, except that now the results for Method C have been additionally superimposed (in blue). Our alternative approach reduces the under-representation at the low degree end, and at the same time also alleviates the over-occurrence of the high degree TFs. This improvement is quantified in terms of the root mean-squared error (RMSE), which shows a > 4-fold decrease (0.02 for Method C against 0.09 for the default method). Overall, the degree-dependent bias suggested by the red curve (the standard approach of testing TFs one at a time for over-representation) is to a considerable degree suppressed in our method (Fig 10, lower left panel).

**Fig 11:**
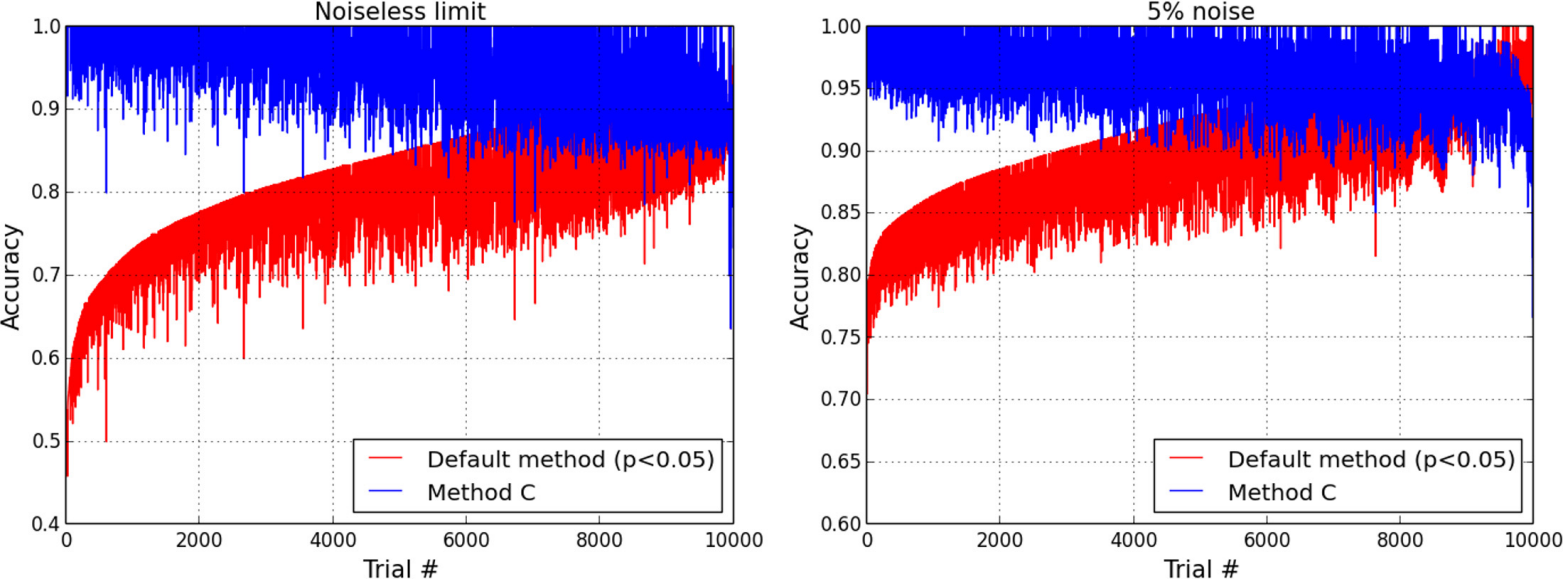
Recovery accuracy for TFs based on simulated trials. Comparison of recovery accuracy values yielded by the default method and iterative search over 104 random trials in the noiseless limit (left) and with 5% misclassification rate added to the binarized differential expression profile (right). The trials have been sorted in decreasing order of the *difference* between the accuracy values (Method C—default).

We find that the strategy of identifying the top-ranked combination of TFs also leads to improved accuracy in the recovery of TFs, i.e. it recapitulates the input set of TFs better. This is seen from Fig 11 (left panel), which plots the accuracy values as a function of the trial number. The trials have been reordered to highlight the difference between the accuracy values (Method C—default), which decreases from left to right. For the RegulonDB graph [26], in 99.2% of the trials Method C achieves improved performance compared with the default methodology (as opposed to only 0.4% going in the opposite direction), and yields significantly higher accuracy values overall (Wilcoxon signed-rank test p-value = 0). This result is fairly insensitive to the choice of the cutoff for assigning statistical significance in the standard test, and is reproduced over a range of threshold values (0.01-1e-6).

We further considered the addition of noise, mimicked by introduction of a small proportion of misclassified genes, wherein 5% of genes are randomly selected from the network and reassigned to the opposite category (differentially expressed v/s unaltered). As shown by the profile of occurrence frequencies in Fig 10 (upper right panel), the deviations from the nearly flat input distribution are largely suppressed upon application of Method C. For example, at p < 0.05 cutoff, the RMSE decreases from 0.072 (standard approach) to 0.022 (Method C) (Fig 10, lower right panel). Pair-wise comparison of the accuracy values of the recovered TF sets, displayed in Fig 11 (right panel), shows that in 91% of trials Method C improves on the output of the default significance test, and also yields considerable improvement in the number of trials in which unit accuracy is obtained (736 v/s 20, out of 10^4^ trials). Thus, once again we find that the earlier trends are essentially reproduced here, and it is evident that a small distortion of the idealized differential expression pattern has minimal impact on the validity of the earlier result. (Similar trend is also seen at 10% noise level: Method C yields improved accuracy in 71% of trials relative to the default method.) Taken together, the preceding results make a case for the utility of the combinational approach as an improved and logically sensible alternative for generating less biased, biologically meaningful hypotheses for altered TF activity from global expression data.

### The combinatorial approach applied to *M*. *tuberculosis* TFOE data demonstrates marked improvement in recovery of the causal TF

As another independent validation of the methodology presented here, we applied both the standard approach and the combinatorial search to microarray profiles for *M*. *tuberculosis* coming from recently published TF overexpression experiments [63], to see how well the network based inference alone (disregarding TF expression change) can recover the causal upstream TF. Out of 78 TFOE experiments where the over-expressed TF was present in the reference regulatory network, we noted that in only 48 cases, the target gene set of the TF had a non-zero overlap with the differentially expressed gene set (identified by imposing a fold change cutoff of 2.0). This is merely indicative of the incomplete nature of the background network used [47,48], and illustrates a limitation which can be expected more generally when carrying out system-level analysis, on which scale the availability of interaction information is unlikely to be complete and altogether accurate. Our comparison shows that the evaluation of each TF separately by Fisher's exact test is able to identify the causal TF in only 21 (44%) cases (at the maximum acceptable p = 0.05 cutoff); in contrast, the TF combination arrived at by Method C contains the causal TF in 32 (67%) cases, representing ≈ 50% improvement. In fact, there are 16 experiments in which Method C uniquely identified the over-expressed TF, which by itself fails to show significant enrichment when tested against the *full* set of differentially expressed genes (this may be contrasted against only 5 cases where the opposite is true). We further wanted to assess whether a simple ranking of the individual over-abundance p-values, regardless of whether they are deemed significant or not, is able to reveal the correct TF. In none of the 16 cases mentioned above, the over-expressed TF is found ranked first in the sorted list. It is interesting to additionally observe that in 10 of the 16 cases, not only is the causal TF not over-represented individually, but it even fails to show up among the top *k* sorted TFs, where *k* denotes the size of the corresponding non-redundant TF combination identified by Method C. Trends similar to those just described are also found for other choices of the cutoff used to identify differentially expressed genes (Table 3). These differences in outcome once again underscore the limitation of assessing TFs one at a time, even in application to single TF OE profiles, with scope for improvement being suggested by the combinatorial perspective explored in the present study.

**Table 3:** Comparison of combinatorial approach (Method C) with Fisher’s exact test for individual TFs on *M*. *tuberculosis* TF overexpression (OE) profiles.

Criterion for significant differential expression →	Fold change (FC) > 2	FDR < 0.05	FDR<0.05 and FC>2
# valid conditions (OE TF present in background network and target set overlaps with DE genes)	48	36	29
# conditions in which OE TF is individually enriched at p<0.05	21	16	18
# conditions in which OE TF is present in group identified by Method C	32	21	20
# conditions where default test *uniquely* identifies OE TF (at p<0.05)	5	1	2
# conditions where combinatorial approach *uniquely* identifies OE TF	16	6	4
# conditions out of the above where OE TF is ranked first in list sorted by p-values (occurs among the top *k* sorted TFs, *k* being size of Method C output)	0/16 (6/16)	1/6 (5/6)	0/4 (2/4)

Results for three different criteria to identify significantly differentially expressed genes are summarized.

## Discussion

Rewiring of transcriptional regulatory networks under different perturbations is an important problem that needs to be understood on the systems level. Several methodologies have been proposed in the literature to interpret data derived from high-throughput profiling experiments in terms of enriched biological functions or over-abundant proximal regulatory elements [64–67]. The particular approach which has been the starting point for the present work involves reducing the case versus control comparison of large scale gene expression to a binary profile with a step-like threshold, where each gene is classified as either differentially expressed or unchanged according to the result of some suitable chosen statistical test applied to the expression values. Pathways or DNA *cis*-regulatory motifs which over-occur in the differentially expressed subset are then inferred based on the null hypothesis that the significantly altered genes are randomly distributed over the genome, and have no statistical association with the functional gene set under consideration. We note that the results of this approach are dependent on the threshold deemed significant for calling a gene differentially expressed, and would in general vary as the dichotomous assignment changes according to different choices of this confidence p-value. Other methods proposed in the literature work instead with the unfiltered expression values directly and are less sensitive to this subjectivity. For example, the popular methodology GSEA [68] computes enrichment scores for gene sets (such as pathways or targets of regulatory motifs) starting with an ordered list of all genes ranked according to some measure of expression change (e.g. t-test score), to identify gene sets whose members show concordant changes in expression between two phenotypes. Furthermore, GSEA and its related offshoots [68–70] use a permutation-based approach of randomly reassigning sample labels to generate a null distribution of scores, which thus differs from the negative control defined for the hypergeometric test that assumes a random binomial distribution of gene labels to estimate statistical association. While Fisher’s exact test applied to binarized data, which is the basis for the present approach, has its limitations, this sort of test is in fact used quite widely in enrichment analyses, as illustrated by the examples mentioned in the introductory section [32–43,67]. Thus, the motivation for the present work is quite justified, and the relevance and utility of the alternative approach we have proposed and explored here should be assessed against the backdrop of these other currently existing tools for transcription factor analysis, all of which are essentially based on application of either the hypergeometric test or some close variant thereof.

It is suggested here that an equally acceptable hypothesis for differential regulation can instead be arrived at by seeking a group of TFs which is *collectively* most predictive for the altered large-scale expression. This approach identifies a candidate set of TFs that is overall distinct and *not* trivially obtainable from a sorted list of TFs arranged in ascending order of their *individual* over-representation p-values. This alternative methodology presents the practical difficulty of having to deal with a solution space whose size grows exponentially with the overall number of TFs in the network. Therefore, greedy heuristics were proposed and their efficacies compared. Our comparison (based on application to *E*. *coli* microarray data) between two quadratic methods for estimating an approximate solution, in particular, holds some relevance going beyond the problem studied here. Many machine learning approaches to classification, e.g. logistic regression, involve identifying a small subset of discriminative features from a high-dimensional, more diverse feature vector. Linear methods which sequentially add features to a growing set based on the discriminative performance of each individual feature are usually adopted for this purpose (e.g. [71]). Our analysis of Method C suggests an alternative take on feature selection which might lead to improved classifier performance, especially when dealing with a large number of potential features. We reiterate that this procedure is distinct from the other iterative method (Method B) which is analogous to the set cover heuristic [72].

The current work represents one approach to address the question of differential TF activity. A few other network reconstruction methods to come up with candidate regulators have been proposed before. The causal reasoning model [73] also seeks to suggest an upstream cause of transcriptional changes, and, based on a scoring function, selects one among multiple competing hypotheses which could act indirectly to regulate expression through downstream signaling. Our setting in its current form only deals with direct regulatory interactions between TFs and target genes, but could well be extended to similarly suggest indirect hypotheses lying further upstream. For example, if TF A regulates the transcription of TFs B and C, then perturbing A would have the effect of changing expression of genes targeted by B and C as well as the targets of A. All three TFs could in principle be identified in the optimal combination yielded by our methodology; thus, a causal role for A could be suggested on the basis of this. It is further noted that the combinatorial method explored here can also handle differential expression data arising from perturbing activity of multiple regulatory pathways which may be completely independent, i.e. not involving any cross-talk. Scoring of such combinations of hypotheses is only alluded to in [73], which can only suggest one upstream node (hypothesis) at a time. Another somewhat different approach is based on the construction of minimum spanning trees to connect all differentially expressed genes on a background functional gene network [74,75]. The intermediate connecting nodes (genes) lying on the resulting Steiner tree become candidates to explain the expression changes. This method also makes use of network information, and in particular, requires the inclusion of all the genes in the queried list in the reconstructed tree. Given that large scale data can be noisy with false positives, it is somewhat doubtful if this is appropriate, with the possible caveat of ending up with spurious candidate markers. Moreover, the absence of a properly defined control (e.g. comparison with randomized data) raises the question of reliability of this approach and significance of the identified candidates. In contrast, our method, being based on statistical association (p-values), provides a robust and sound procedure that is more apt for handling noisy genome-scale data.

A possible direction to extend the present work in would be to incorporate the principle of parsimony. From the point of view of practicality, a simpler hypothesis (i.e. a smaller number of inferred TFs) might be more favorable, and so, instead of the global *minimum*, an *optimal* subnetwork could be sought which strikes a judicious balance between the TF set size and the fit to the data (over-abundance p-value), analogous to Bayesian/Akaike information criterion for parameter estimation in multivariate regression [76]. This could be implemented in the iterative approach by, e.g., requiring that every additional TF incorporated into the growing solution provide some minimum reduction in the overall p-value. Once the point is reached beyond which further addition of TFs does not produce substantial improvement, the algorithm could be terminated. Since we wanted to keep the analysis presented here fairly general, this additional constraint was not imposed, as it would require introducing a user-defined parameter which decides the balance between parsimony and fit, and the outcome of the procedure would then depend on the numerical value of this additional parameter.

Finally, we note that the present methodology could be revised to incorporate combinatorial effects of TFs. This is especially relevant in the case of eukaryotes and higher organisms where cooperative synergistic interactions between pairs (or even larger groups) of TFs are quite common in the transcriptional control of gene expression [77–80]. In particular, if a TF requires co-binding of other regulators to nearby DNA sequences to modulate the rate of transcription of its gene targets, then an analysis based on the entire set of binding sites of that TF, as identified e.g. from enriched peaks in a ChIP-seq genome-wide binding profile [16], may not give correct results, as many of those binding sites may not translate into bona fide causal regulatory interactions. Thus, the use of gene sets which are co-targeted by multiple TFs (i.e. regulatory ‘modules’) as building blocks, instead of the target sets of individual regulatory motifs as has been followed here, might provide more realistic hypotheses for discriminative regulatory subnetworks. The results of such an analysis could in fact even be leveraged to reveal novel TF-TF combinatorial effects. These ideas are currently under investigation.

In summary, we have proposed a general, unbiased, and logically natural methodology to come up with a set of differentially active regulatory factors implicated by large-scale differential gene expression data. It avoids the somewhat artificial breaking up of the problem that happens when testing each regulator separately. By effectively boosting regulators that may individually fail to show enrichment, our strategy holds out the possibility of revealing biologically important regulators. Such cases might be missed by many standard approaches that all share the common feature of assessing each TF separately for overlap against the full list of altered genes. This perspective should be of immense utility in contributing to a better mechanistic understanding of the global transcriptional remodeling underpinning cellular adaptation, or dysfunction.

## Materials and Methods

### Re-examining the default approach to identifying perturbed regulators in the noiseless limit with simulated profiles

We assume a noiseless setting in which a subset of transcription factors (size ranging from 5 to 20) is randomly chosen for differential activation, and *all* their direct targets are assigned to the differentially transcribed gene set. Overlaying this idealized differential expression profile on the transcriptional regulatory network, we first assess how well the Fisher's exact test applied to individual TFs recovers the original subset. For this example, we have made use of the regulatory network for *E*. *coli* retrieved from the RegulonDB database (Release 8.6, dated 4-11-2014), which comprises 4055 empirically validated regulatory interactions spanning 197 transcription factors and 1934 genes, covering nearly half of the coding genome [26].

**Fig 12:**
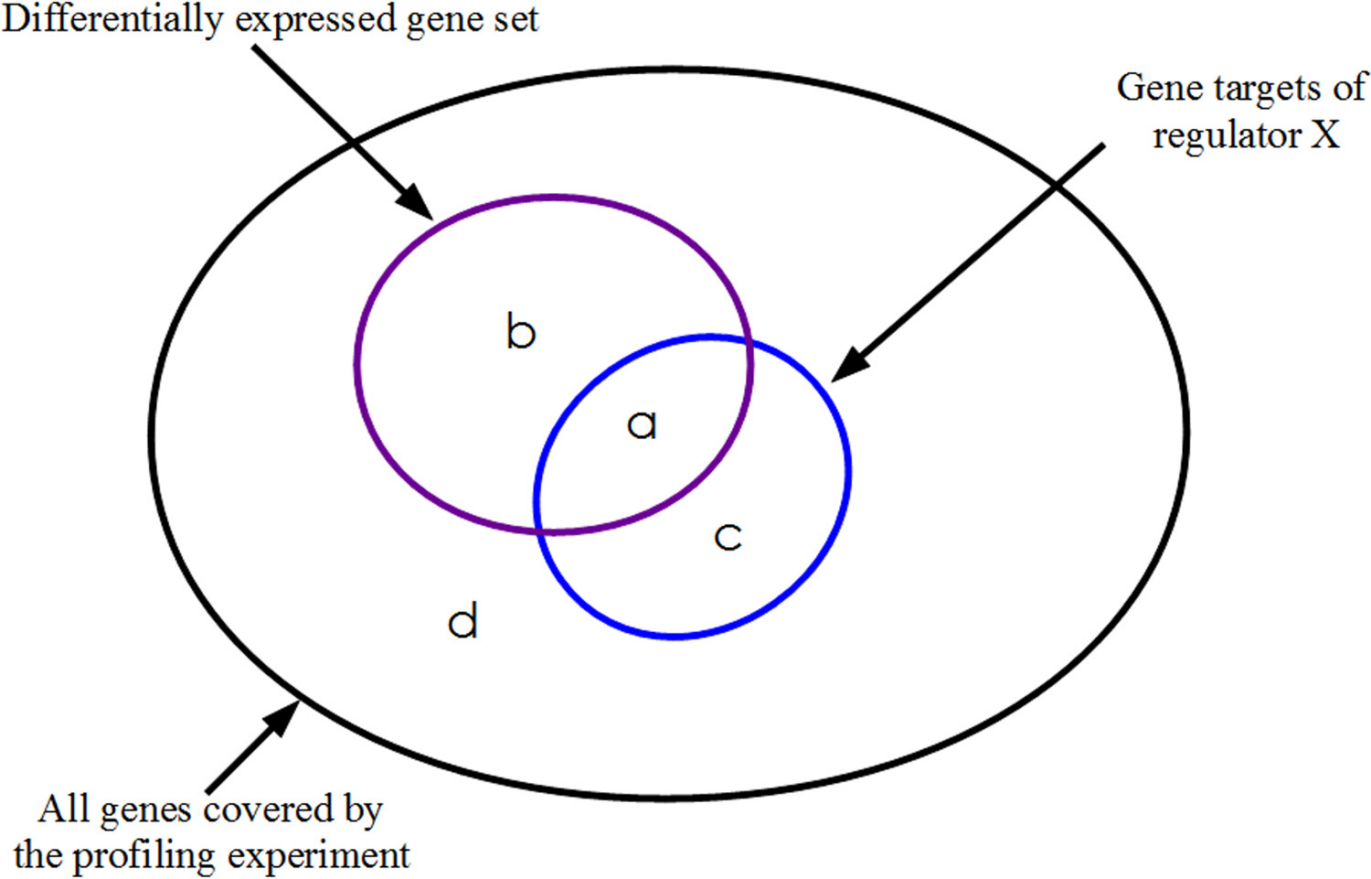
Transcription factor enrichment from large scale differential gene expression data. Graphical representation of the entries a, b, c and d in the 2x2 contingency table on which the computation of the enrichment p-value by Fisher's exact test is based. This test evaluates the probability that the size of the observed overlap (a) is consistent with random distribution of the differentially expressed subset.

We have applied one-sided Fisher's exact test [81] to identify TFs significantly associated with the differential gene expression. This test of significance yields the probability that an overlap between the targets of a TF and the genes with altered expression at least as large as the one observed can be explained by chance (Fig 12). In every trial, the over-representation p-values have been separately obtained for all regulators having non-zero overlap with the differentially regulated gene set. The raw p-values obtained in this manner are adjusted by applying Bonferroni correction [82] to account for multiple hypotheses testing. For different choices of the p-value threshold for significance, statistics for occurrences of all the TFs have been obtained over 10^4^ random trials.

The recovery of the input set was assessed in terms of the prediction accuracy [83], calculated as the fraction of correct classifications out of the total test set (all TFs having at least one differentially expressed target each). If *TP* denotes the number of true positives, i.e. the TFs correctly recovered from the input set, *P* stands for the total number of TFs present in the input set, *TN* is the number of TFs which are not part of the input and are not found in the enriched set, and finally *N* denotes the complement of the *P* subset, then the accuracy is given by (*TP*+*TN*)/(*P* + *N*).

Thus, for example, if at the p < 0.05 level the enriched set of TFs is found to be identical to the input set, the accuracy assumes its maximum value of unity, and any mismatches would result in a lowered accuracy, restricted to the range 0 to 1.

### Identification of key regulators underlying differential expression by evaluating combinations of TFs

Our objective has been to identify a group of transcription factors that best accounts for observed differential gene expression. An exhaustive search for such a combination is usually not computationally tractable.

A greedy search that iteratively builds up an approximate solution by making the best local choice at every step is routinely sought in problems of this nature. We have evaluated three variants of a heuristic procedure for obtaining an approximate solution (flowchart in Fig 4), one of which has linear dependence of the number of p-value computations on the number of overlapping TFs, and two other alternatives which have quadratic runtime.

The simplest procedure involves first ranking and ordering the TFs in accordance with their individual overlap p-values, and then, starting from the top-ranked regulator, sequentially adding the next TF from this sorted list until there is no further improvement in the combined p-value that is evaluated for the union of direct targets. We denote this heuristic as Method A. As this scheme makes use of the original prioritized list made at the initialization to select a TF at every update, only one p-value is computed at every subsequent step, giving an O(N) dependence of the number of p-value computations on the size of the sorted list (i.e. on the number of relevant TFs, N). Given that at each step, the best available choice is made, there is no guarantee that the converged solution represents the global minimum.

The efficacy of the above simplistic method is assessed against two other approximations, both of which involve generating a prioritized list of TFs at every step in the iteration. Thus, these approaches would have a time complexity of O(N^2^). Method B is analogous to the greedy search proposed for obtaining an approximation to the set covering problem [72]. Starting with the best single TF, as in Method A, at every subsequent step, one additional TF is added to the growing solution. In order to make this choice, we apply the criterion that, the next TF chosen is the one which has the best overlap p-value evaluated on the *remaining* subset of altered genes, i.e. those which are not already covered by the running set of TFs built up thus far. The search is stopped when no further addition of a TF yields a reduction in the combined p-value.

A second O(N^2^) procedure, Method C, is additionally implemented. This is similar to Method B, in that a rank-ordered list of the remaining TFs is generated at every update. However, now we employ the criterion that the next TF chosen is the one which, *when added to the already built up (running) solution*, gives the best *combined* p-value. Once convergence is reached such that no additional TF provides further improvement in the collective p-value, the progressive search is halted, yielding a candidate solution.

### Comparative analysis of approximations on published microarray data

We have performed a comparative analysis and benchmarking of the three approximations defined above on a set of microarray expression profiles downloaded from GEO [84] describing responses of wild-type *E*. *coli* to various stress conditions. Expression datasets associated with previous publications [49–52] for the following stress conditions for *E*. *coli* were downloaded: heat shock (45°C for 10 minutes) and stationary phase; pH 5.0 and pH 8.7; non-ionic (sucrose) and ionic (NaCl) osmotic stress; and 1 ug/ml norfloxacin exposure, with the corresponding GEO accession numbers being GSE12190, GSE4511, GSE15534 and GSE6836 respectively. For each experiment, a set of genes differentially transcribed in a test v/s control comparison were first identified by using the accompanying GEO2R tool [85], uniformly applying an FDR threshold of <0.1% and a minimum fold-change criterion of 2.0 across all the datasets. By integrating with the RegulonDB compilation of regulatory interactions, each of the seven gene sets was then used to infer a discriminative combination of TFs by each of the three iterative methods A, B and C.

A similar comparison among the three methods has been carried out on a larger compendium of normalized expression profiles for *E*. *coli* retrieved from the Many Microbes Microarrays Database (M3D, Version 4, Build 6) [53]. For the 466 experiments representing various perturbation conditions in M3D, the RMA-processed log2-transformed expression values across all the conditions were first normalized by converting them to gene-wise z-scores. These quantify how much the expression of a gene in any particular condition deviates from the baseline defined by its mean over all the conditions. Applying a cutoff of abs(z-score) > 2.0, for every condition, genes which show ‘abnormal’ expression with respect to their global average were identified. Thus, the approach we have followed here does not involve comparing the expression values on each array with a common control condition. Instead, the control for every gene is set by its global average.

### Ruggedness of search space, and iterative search v/s steepest descent v/s stochastic search

Ruggedness of a combinatorial search space is normally defined in terms of the number of local minima accessible by steepest descent search [54–57]. Steepest descent for the current problem has been implemented in the following manner: starting from a randomly selected initial configuration, at each update, the current state (overlap p-value) is compared with all its neighboring configurations, which each differ by the addition or exclusion of a single TF. If a neighboring configuration with a lower overlap p-value is found, then it is chosen as the new solution. This updating is continued until no further improvement in the p-value is obtainable by moving to a neighboring state. For random starting state, local minima with larger basins of attraction are more likely to be reached. Summary statistics for the ruggedness was obtained by running gradient descent search 2000 times from different starting configurations, sampled uniformly, and the number of distinct overlap p-values obtained upon convergence was tracked as a function of the number of runs. This exercise was repeated for all the seven GEO profiles.

We also implemented random walks in the search space that provide an additional measure for ruggedness [54]. Each trajectory was initiated at a randomly chosen initial state, and at every step, a neighbour of the current state (differing by a single TF) was arbitrarily chosen. Pearson correlation coefficient between log p-values as a function of the number of steps was estimated by averaging over 5000 such trajectories. This autocorrelation function is expected to decay over a length scale set by the smoothness of the search space.

As an alternative approach to seeking the global minimum in a complex solution space, we have also implemented a simple formulation of simulated annealing (SA). This heuristic represents one among several different optimization techniques which contain a stochastic component, allowing to overcome barriers in the search space and to avoid getting trapped in local optima [55,58–61]. SA modifies steepest gradient search by permitting locally non-optimal updates which can increase the p-value (the objective function here), the frequency of which is controlled by an effective temperature T. The numerical value of T modulates the probability that an update resulting in a change in the (log) p-value is accepted, via the following formula:

$$P_{accep}(T)=\min\;(1,\;e^{-\frac{\Delta (\log\;p)}{T}})$$

The effective temperature is gradually decreased as a function of the number of steps according to a suitably chosen annealing schedule. In the T → 0 limit, SA thus reduces to the usual gradient descent search. In order to circumvent issues related to optimization of the choices for temperature and annealing schedule tailored to the geometry of the search space [59], we have adopted a simplified approach by repeatedly running SA over a range of timescale settings and picking the best run in terms of the final p-value attained. The starting temperature T_i_ has been set by requiring that the initial average probability for accepting non-optimal jumps is 0.8 [58,60]. An exponential annealing schedule [61] has been adopted, parametrized by a fractional factor r which controls the rate of temperature reduction according to the formula T_n+1_ = rT_n_ at the *n*-th step. Given a final temperature T_f_ (here kept fixed at 0.01 across all runs and examples), the total number of steps in one run is directly related to r by the relation N_SA_ = log_r_(T_f_/T_i_). We have repeated the search procedure for N_SA_ values ranging from 5N to 25N in steps of 5N, and 10 runs starting from different random initial combinations were simulated for each choice of N_SA._ The best p-value (and corresponding TF group) attained during the course of every run was recorded. Then, starting from this configuration, gradient descent search was run at the very end to ensure convergence to the nearest minimum in the search space. The lowest log p-value obtained across the full set of runs was finally taken to represent the solution yielded by the SA approach, and used as an independent benchmark to assess the results of the iterative methods.

### Objective assessment of significance of the obtained TF subnetworks

Our method is bound to yield a TF combination, regardless of the signal-to-noise ratio of the binarized differential expression profile. One therefore needs to have an independent means of ascertaining whether the obtained TF subset is a meaningful hypothesis, and not just an artefactual outcome of the search. In order to do this, we define a 'negative control' distribution of p-values as follows: *n* genes are randomly selected from the background network and assumed differentially expressed, where *n* is the number of differentially expressed genes in the original profile. The combinatorial search (Method C) is run on this randomized profile and the resultant optimal log p-value is recorded. This exercise is repeated a large number (here, 1000) of times, and a baseline distribution of log p-values is thereby generated. The combinatorial p-value that was computed for the original (experimentally obtained) differential expression profile can then be compared with this negative control distribution by calculating a z-score. A large negative z-score would indicate that the empirical p-value is much more significant than one yielded by a completely randomized profile, providing support for regarding the differential transcription data to have indeed originated from perturbing TFs in the underlying network. In order to justify use of a z-score as a valid metric for comparison, Kolmogorov-Smirnov (K-S) test (implemented in SciPy v0.11.0) is applied to the control distribution of log p-values to assess normality.

### Benchmarking the proposed methodology in the idealized setting

Reverting to the noiseless limit introduced earlier, in every random trial we also run the iterative Method C to obtain a proxy for the TF combination with minimum collective p-value of association. (The choice of Method C follows from our analysis which demonstrates that it is the best among the three iterative methods A-C proposed.) The corresponding distribution of TF occurrences across the 10^4^ trials is then compared with the distribution for the default method which was obtained earlier. The overall deviation of either distribution from the near-uniform input distribution is quantified in terms of the root mean squared error (RMSE), estimated by averaging over all TFs the squared difference between the proportions of trials in which the i-th TF occurs in the input and output sets. A comparison between the lists of the corresponding accuracy values is also made by applying Wilcoxon signed-rank test for significant difference [86] implemented in SciPy v0.11.0.

In order to confirm whether the trends are sensitive to the presence of misclassification (noise) distorting the expression pattern, which is expected in any large-scale experimental dataset, we have revised the earlier deterministic setting by adding noise in the form of misclassification of genes. This has been implemented by randomly selecting a fraction of the genes in the network and reassigning them to the opposite class. Thus, if a differentially expressed gene is chosen, it is reclassified as unaltered, and vice versa. The performance of the two inference methods (default and Method C) was compared over 10^4^ random trials in the presence of 5% and 10% misclassification rate.

### Independent comparison on *M*. *tuberculosis* TF over-expression experimental data

As a final and independent assessment of the presented methodology on a different organism, we have applied it vis-à-vis the standard approach in the context of *M*. *tuberculosis* (MTB) transcription data. Microarray profiles made available as part of a recently published large-scale study [63] were downloaded from [87]. This dataset comprises over-expression phenotypes for 206 MTB TFs. Significantly differentially expressed genes were identified in each experiment based on three different filters: a) minimum expression fold change > 2.0, b) FDR < 0.05, and c) both FDR < 0.05 and fold change > 2.0. This expression change information was integrated with a curated transcriptional regulatory network composed of 91 TFs and 3682 regulatory connections covering 1787 MTB genes in total. This reference network, which merges two independent previously published studies [47,48], was assembled by combining curated experimental evidences with orthology-based mapping from related species (*E*. *coli* and *C*. *glutamicum*). As in the previous analyses, the standard testing of individual TFs as well as the combinatorial approach (Method C) was applied to each gene list. The recovery performance was assessed in terms of the number of times the causal TF (known *a priori*) occurs among the TFs individually statistically enriched at p < 0.05 confidence threshold, relative to the number of times it is present in the TF group yielded by the sequential search (Method C).

## Supporting Information

S1 Fig Coverage of significantly altered genes by the TFs inferred by the default and combinatorial approaches applied to *E*. *coli* expression profiles in the M3D compendium. Z-score threshold for identifying significantly altered genes in every experiment = ±2. Out of 466 profiles in total, Method C covers a larger proportion of genes in 78% of the cases (red points lying above the diagonal), as compared to only 16% where the set of TFs individually associated at p<0.05 level yields higher coverage (blue points).
(TIF)

S2 Fig Benchmarking the iterative methods on M3D data for *E*. *coli*. Comparison of the three heuristics applied to a subset of 198 M3D expression profiles where the manageable numbers of overlapping TFs make exhaustive enumeration possible (z-score threshold for significance of altered expression = ± 3.0). The light blue bars represent the number of times that each of the methods identifies the global minimum (left y-axis). The dark blue bars display the mean rank of the estimated solution in a list of all possible TF combinations sorted according to their combined p-values (right y-axis).
(TIF)

S1 File Python script implementing the sequential search methods A-C. (PY)

S1 Table Coverage of significantly differentially expressed genes. Coverage of significantly differentially expressed genes by the TFs inferred via default and combinatorial approaches applied to *E*. *coli* GEO microarray data, both of which test for the statistical significance of the association of TF target sets with the significantly differentially transcribed subset of genes.
(DOCX)
