# Supplementary material for: From System-Wide Differential Gene Expression to Perturbed Regulatory Factors: A Combinatorial Approach
Source: PLoS One. 2015 Nov 12;10(11):e0142147. doi: 10.1371/journal.pone.0142147 (PMC4642966; doi:10.1371/journal.pone.0142147)
Supplement: S1 Table — Coverage of significantly differentially expressed genes by the TFs inferred via default and combinatorial approaches applied to E. coli GEO microarray data, both of which test for the statistical significance of the association of TF target sets with the significantly differentially transcribed subset of genes. (DOCX) [file pone.0142147.s004.docx]

**S1 Table. Coverage of significantly differentially expressed genes.**

| Expression dataset | # TFs identified by Method C (*and* not individually associated at p < 0.05 level) | # significantly diff. expressed genes covered by Method C | # significantly diff. expressed genes covered by TFs individually associated at p < 0.05 level |
| --- | --- | --- | --- |
| pH 5.0 | 23 (17) | 73/176 | 92/176 |
| pH 8.7 | 10 (4) | 46/92 | 44/92 |
| Norfloxacin | 31 (30) | 61/312 | 30/312 |
| Sucrose stress | 20 (19) | 47/277 | 10/277 |
| NaCl stress | 33 (31) | 63/194 | 48/194 |
| Stationary phase | 33 (29) | 241/2314 | 113/2314 |
| Heat shock | 63 (60) | 576/3139 | 228/3139 |

Table 1 legend: Coverage of significantly differentially expressed genes by the TFs inferred via default and combinatorial approaches applied to *E. coli* GEO microarray data, both of which test for the statistical significance of the association of TF target sets with the significantly differentially transcribed subset of genes.
